# THE IMPACT OF 3 GENERATIONS OF VIOLENCE ON THE MENTAL HEALTH OF COLOMBIAN OLDER ADULTS: COMPARATIVE MULTI-SITE SURVEY

**DOI:** 10.1093/geroni/igad104.3799

**Published:** 2023-12-21

**Authors:** Mark Gabbay, Maria Isabel Zuluaga, gabriel Saldarriaga-ruiz, Erika Montoya, Clarissa Giebel

**Affiliations:** University of Liverpool, Liverpool, England, United Kingdom; Universidad de Antioquia, Medellin, Antioquia, Colombia; Universidad de Antioquia, Medellin, Antioquia, Colombia; Universidad de Antioquia, Medellin, Antioquia, Colombia; University of Liverpool, Liverpool, England, United Kingdom

## Abstract

Endemic violence across Colombia for 70 years has displaced generations of the poorest among Colombian society particularly in (semi)rural and peri-urban settings, approximately (10m- 20% total population). In 2022, 12.5% (1.2m) were older adults living with the health and socioeconomic consequences, losses and life-changing disruptions. Our joint Colombian UK-funded project in Turbo explores ways to co-produce and pilot an intervention to help mitigate impacts older-adults’ mental-health and wellbeing. This survey explores their burden of ill-health and adversity. We compared our 2022 older-adults survey in Turbo ( n=611) with 2016 (n=~0.5m) survey findings from three other Colombian regions with different exposures to ‘La Violencia’. Both surveys measured Cognitive status, Functionality, Autonomy, Depression, Anxiety, Alcohol-use, Well-being, Social support, Loneliness, Nutrition and Maltreatment plus demographics and used probability cluster two-stage sampling to enhance representativeness. Mental health is not a concept used by older adults, ‘depression’ (5-28%) and ‘anxiety’ (4-21%) were lower than anticipated. An unexpectedly high proportion in Turbo respondents had at least some cognitive deficit (62%), much higher than elsewhere (1.3-5.1%). Most respondents were living in significant poverty with low educational attainment, low or minimal income, low nutrition, poor housing and social isolation. Reported elder abuse rates ranged from 4-30% across sites. The survey findings confirm high rates of vulnerability among older adults living with the severe consequences of ’La Violencia’ upon mental-health. Our project co-producing and delivering affordable and sustainable interventions, seeks to improve mental health and wellbeing, self-esteem and belonging, within a society that largely neglects older adults.

